# Targeting Pain Science Education With a Children's Book: A Single Case Experimental Design (SCED) Study With a Sham Comparison

**DOI:** 10.1155/prm/7548771

**Published:** 2025-11-05

**Authors:** Joshua W. Pate, Laura E. Simons, Emily Moore, Jennifer Norton, Erin Turbitt, Arianne Verhagen, Verity Pacey

**Affiliations:** ^1^Discipline of Physiotherapy, Faculty of Health, Graduate School of Health, University of Technology Sydney, Sydney, New South Wales, Australia; ^2^Department of Anesthesiology, Perioperative, and Pain Medicine, Stanford University School of Medicine, Stanford, California, USA; ^3^Department of Health Sciences, Macquarie University, Sydney, New South Wales, Australia

**Keywords:** children's books, conceptual change, pain science education, pediatrics, single case experimental design

## Abstract

**Objectives:**

To assess perceived changes in children's understanding of the pain–damage relationship and the brain's influence on pain following exposure to sham and pain science education (PSE) books.

**Methods:**

A series of single-case experimental designs were replicated across 17 children (8–12 years), with and without chronic pain, over six weeks. Following baseline, participants received a sham book and then a PSE book. The Concept of Pain Inventory (COPI) (total and selected items) and qualitative interviews (child and interviewer) assessed conceptual change and book acceptability.

**Results:**

COPI scores for three targeted items fluctuated but generally increased after the PSE book for both groups, suggesting improved understanding. Discrepancies between COPI responses and interview data occurred in 88% of children, indicating varied perspectives on the nature and extent of conceptual change. The PSE book was found to be acceptable and was preferred by most children.

**Discussion:**

Children's understanding of core pain science concepts can improve with targeted, book-based PSE. The dynamic nature of conceptual change and observed discrepancies between quantitative and qualitative assessment methods underscore the value of using multiple approaches to evaluate learning in pediatric PSE.

## 1. Introduction

Chronic pain (> 3 months) in children is both prevalent and burdensome. Point prevalence estimates range from 11% to 54% [1]. Pediatric chronic pain begins to emerge in late primary school (ages 8–12 years) and has been shown to reduce quality of life [2], increase disability [3], increase school absence [4], and predict chronic pain later in life [5]. Pain science education (PSE) is an important component of multidisciplinary treatment for children with chronic pain [6]. By facilitating a shift towards a nuanced, biopsychosocial understanding of pain, a primary goal of PSE is to decrease the threat of pain, ultimately leading to greater willingness in children to engage in physical rehabilitation and improve functional outcomes [7–10]. PSE is based on conceptual change theory [11] where existing knowledge frameworks are restructured, rather than simply adding new information. Key PSE learning outcomes address ideas such as the relationship between pain and damage and the influence of the brain on pain [12, 13].

A child's understanding of pain science has been cross-sectionally assessed in previous research using qualitative and quantitative methods [14, 15]. However, it is currently unknown how to best assess when a change in understanding of pain has occurred over time and in response to specific educational inputs. Current research suggests that educational strategies for children with chronic pain should be individualized using age-appropriate language, addressing specific misconceptions identified in an individual child, and employing different modalities like storytelling or interactive activities [16]. Assessing a child's concept of pain can theoretically enable targeted PSE using the 14-item Concept of Pain Inventory (COPI) [15]. For example, if a child responds “unsure” to the item “*You can feel a lot of pain even when an injury is small,*” the child could be prompted to explore an educational resource addressing that particular idea. Comparing changes in COPI item scores over time to repeated qualitative interviews could provide new insights about the process of conceptual change in a child's understanding of pain.

No previous research has assessed changes in a child's concept of pain over time using both qualitative and quantitative methods in relation to a book-based PSE intervention. Using assessment tools like the COPI, neither individual-level nor item-level conceptual changes have been researched longitudinally to date. Therefore, our primary aim was to assess perceived change in understanding about the relationship between pain and damage and about the influence of the brain on pain following exposure to sham and PSE children's books. Our secondary aim was to assess the acceptability and usability of a PSE children's book.

## 2. Materials and Methods

### 2.1. Design

We used a single-case experimental design (SCED) to compare child reports over time from an interviewer, a child's verbal responses, and their survey responses. We assessed differences comparing a brief PSE children's book to a sham educational children's book during a 6-week period. This study was approved by the University of Technology Sydney Human Research Ethics Committee (REF: ETH21-5786).

### 2.2. Participants (Subjects)

We invited two groups of English-speaking children, aged 8–12 years, living in Australia to participate in our study: (1) children with self-reported chronic pain and (2) children who self-reported to be pain-free. We excluded children deemed unsuitable by their parent (e.g., severe cognitive impairment). Parents were provided with information about the study and an informed consent form to sign. When children were eligible and included, parents were advised to be present during their child's participation, but not to prompt or correct the responses of their child.

A recruitment target was set of 10 children in each group after consultation with a statistician. The SCED design affords the opportunity for a small sample, as each individual serves as their own control [17, 18]. We enrolled children with and without chronic pain to explore feasibility and potential moderation by pain status. The study was not powered for between-group effects and so any comparable patterns may reflect ceiling effects, shared mechanisms, or measurement limitations.

### 2.3. Procedure

**Figure 1:**
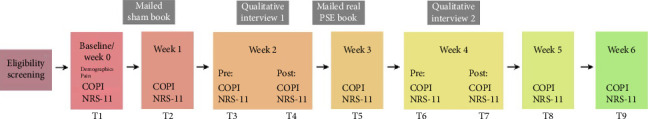
Flow of participants through the nine time points in the study.

Children with chronic pain were recruited via private health professionals handing out information sheets. Children without pain were recruited via a social media post. After consent was obtained, children completed the baseline survey and were then mailed a physical copy of the first book (sham) and emailed a link to a video reading of that book.  Figure 1 shows the flow of child participants through the study. A qualitative interview was conducted via Zoom during Week 2. Families were then mailed the second book (real PSE book) and emailed a link to a video reading of that book. A second qualitative interview was conducted during Week 4. Questionnaires were administered at baseline, weekly for 6 weeks as well as one directly after the two interviews, a total of 9 time points. To increase adherence, the following incentive was used: “*For every participant that completes the surveys and 2 interviews, a donation of $25 will be made to a children's hospital on their behalf, to contribute to future pain-related research.*”

#### 2.3.1. Sham Book

This book (titled “Uncle Ozzie Nailed It” to match the real PSE book) was developed by JWP for the purpose of this study and has the same design features as the real PSE book (i.e., it is a brief, colorful, illustrated, rhyming, developmentally appropriate children's book that also mentions the human brain). The sham book does not address a PSE learning outcome; instead, it focuses on how to use a pain intensity rating scale. An example stanza is as follows: “*Imagine the worst pain ever, well, that's the highest score; ‘10 out of 10' is when the pain feels SO BAD you can't have more!*”

#### 2.3.2. Real PSE Book

In a series of five children's books that author JWP developed [19], the first book (“Zoe Zoppins Nails It”) addresses the idea that “pain does not mean my body is damaged.” At the time of this study, an early draft version of this PSE book (then titled “Uncle Ozzie Nailed It”) was available for testing. An example stanza is as follows: “*You can feel sore when you're safe, no doubt! WITHOUT damage inside or out.*”

At the time of registering the study protocol (https://osf.io/p8n6u/ 8 October 2021), both books underwent expert review from 4 external independent pediatric pain researchers and 2 independent pediatric pain clinicians to ensure the accuracy of the science education and likely appropriateness of this as a pediatric interventional resource. The books were then piloted with 3 children independent of the study aged 8, 10, and 12 years to informally confirm that the language was age appropriate via verbal feedback. Minor grammatical changes, and the position and size of some illustrations, were changed based on feedback.

#### 2.3.3. Blinding

Children, and interviewers (Emily Moore and Jennifer Norton), were blinded to the sequence of the sham (always first) and the real PSE book (always second). The sequence was not randomized based on statistical advice because it is hypothesized that knowledge and beliefs do not return to baseline levels after learning this PSE concept. This fixed order also allowed for a consistent comparison of the PSE book's impact relative to the sham condition across all participants. The two coders of each interview transcript (shared among Emily Moore, Jennifer Norton, and Erin Turbitt) were also blinded to whether the transcript they were assessing was related to the sham book or real PSE book. The success of blinding was evaluated after each step of involvement, as the percentage was correct when asked to guess.

### 2.4. Measures (Instrumentation)

#### 2.4.1. Demographics

The following demographic variables were collected at baseline: age, gender, postcode (for index of relative socioeconomic disadvantage (SEIFA) decile/10), chronic pain status, chronic pain duration, and chronic pain location. One further question was asked about recent (< 3 months) experiences that may affect a child's concept of pain, such as pain, injuries, medication, and medical interventions.

#### 2.4.2. COPI

All children were asked to fill in the COPI. This is a 14-item tool assessing concept of pain with a total score (range) from 0–56. Items are rated on a 5-point scale from 0 = *strongly disagree* to 4 = *strongly agree*. Items are summed to derive a total score, with higher scores reflecting knowledge and beliefs more closely aligned with contemporary PSE. The COPI has been shown to have acceptable internal consistency (Cronbach's alpha = 0.78) and moderate test–retest reliability in children [15]. Items 7, 10, and 14 (7, “*You can feel a lot of pain even when an injury is small*”; 10, “*The brain can make pain better or worse*”; and 14, “*The brain processes lots of details before you feel pain*”) were selected for the item-level analysis because these items directly aligned with the PSE book focus and are addressed in this book.

#### 2.4.3. Pain Intensity

All children were asked to provide their typical and current pain intensity rating on a standard validated 11-point numeric rating scale (NRS-11) [20, 21] from 0 = “*no pain*” to 10 = “*most pain possible*.”

#### 2.4.4. Pediatric Pain Screening Tool (PPST)

To further describe the sample of children with chronic pain at baseline, the PPST assessed risk of poor pain-related outcomes [22]. It contains two subscales: physical and psychosocial, with eight agree (1)/disagree (0) items, and one 5-point Likert item: “*a lot*” (1), “*a whole lot*” (1), “*not at all*” (0), “*a little*” (0), and “*some*” (0). PPST summed total scores range from 0 to 9. Total scores of 0–2 are considered “low risk” and psychosocial subscale scores of 3 or more are considered “high risk.” The tool has adequate test–retest reliability at 2 weeks (ICC = 0.75) in a mixed childhood chronic pain sample [22] and has also been tested in children with headaches [23] and sickle cell disease [24].

#### 2.4.5. Anchoring Question for Perceived Conceptual Change

All children were asked at the beginning of the final (9^th^) survey to anchor the perception of conceptual change in understanding of pain: “*Compared to before I participated in this study, I have a different understanding of how pain works.*” This item was scored from 0 (*strongly disagree*) to 4 (*strongly agree*), and so higher scores indicate higher perceived conceptual change.

#### 2.4.6. Acceptability and Usability of the Books

All children were asked at the beginning of the final (9^th^) survey to self-report their frequency of reading the two books (survey questions were blinded as “1^st^ book” and “2^nd^ book” to children) and watching the video readings of the books that were emailed at the same time as the hardcopy books were mailed out. During the 2^nd^ interview, children were also asked about acceptability and usability.

#### 2.4.7. Interviews

All children were involved in two qualitative semistructured interviews via Zoom (at Week 2 and Week 4). Supporting information 1 contains the qualitative interview methods including drawing tasks, in addition to the codebook and topic summaries relating to the purpose and definition of pain.

### 2.5. Data Analysis

Data were entered and analyzed with a complete case analysis using SPSS v27.0 (SPSS Inc, Chicago, IL). When frequencies of descriptive data were not normally distributed, we presented these as median (IQR). To answer the primary aim, we first calculated a Pearson correlation coefficient between the anchoring question score in Week 6 and the overall change in total COPI score from baseline to Week 6. For the correlation coefficient, we used the following cut-off values: *r* < 0.4 = weak correlation; 0.4 < *r* < 0.7 = moderate correlation; and *r* > 0.7 = strong correlation [25]. We then evaluated change for the three COPI items selected for item-level analysis, visually analyzing and reporting changes over the 6 weeks. Following each interview in Week 4, the blinded interviewer was surveyed about whether they perceived that the child's concept had changed regarding the relationship of pain and damage and the influence of the brain on pain (yes/no). During coding, the blinded coders collated the child's self-report of conceptual change during 2^nd^ interview in Week 4. These data were entered into a summary table alongside the item-level analysis.

For the secondary aim, we calculated frequencies and analyzed related interview data using codebook thematic analysis [26]. We a priori developed a preliminary codebook based on the interview guide and our theoretical knowledge and clinical expertise. The codebook consisted of codes (data labels) and code descriptions. As we applied the codebook to analyze the data, we iteratively added codes and added exemplar quotes to the codebook to ensure the codebook was applied consistently across 3 different coders. Codes were then grouped into topic summaries.

## 3. Results

### 3.1. Participants

#### 3.1.1. Demographics

Of the 20 children who enrolled, consented, and completed the baseline questionnaire, 19 completed their first interview (Week 2) and 17 completed their second interview (Week 4): 9 children with no chronic pain and 8 children with chronic pain were included in the analysis. These 17 children completed all 9 surveys.  Table 1 shows the demographics of the child participants in each group. There were no large, obvious differences between groups.

#### 3.1.2. Pain Intensity

At baseline, median (IQR) typical pain intensity in the past week was 0 (0–2) for children in the “no pain” group and 5 (4.5–5) for children in the “chronic pain” group. Median (IQR) current pain intensity was 0 (0 to 0) for children in the “no pain” group and 4 (1.75–5.25) for children in the “chronic pain” group.

Pain intensity ratings fluctuated for children throughout the study period for both children with and without chronic pain. Typical pain intensity ratings fluctuated by 0–6 points over time for children in the “no pain” group and fluctuated by 1–6 points for children in the “chronic pain” group. Current pain intensity ratings fluctuated by 0–3 points over time for children in the “no pain” group and fluctuated by 1–8 points for children in the “chronic pain” group.

#### 3.1.3. Blinding

After the first interview, the blinded interviewers were 57% correct when asked to guess which book was read first (sham). After the second interview, the blinded interviews were 67% correct when asked to guess which book was read second (real PSE book). One participant accidentally unblinded the book during the 2^nd^ interview. The two blinded coders were 76% and 75% correct when asked to guess which book the transcript related to for the first interview (sham book), and they were 41% and 50% correct regarding the second interview (real PSE book).

### 3.2. Conceptual Change in Understanding of Pain

#### 3.2.1. Total COPI Scores

**Figure 2:**
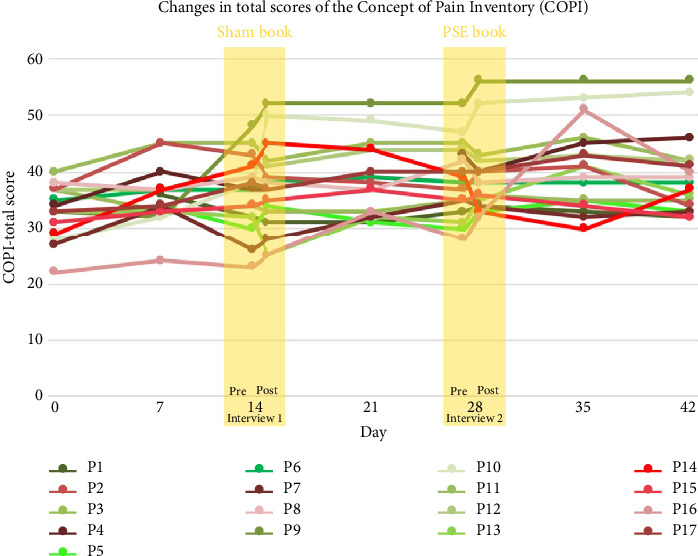
Individual-level changes in total COPI scores over time. Note: children reporting chronic pain were colored with shades of red, and children reporting no pain were colored with shades of green.

At baseline, the median (IQR) COPI score was 34 (31.75–37) for children in the “no pain” group and 32 (28.5–34.75) for children in the “chronic pain” group. At 6 weeks, the median (IQR) COPI score was 38 (35–42) for children in the “no pain” group and 38 (33.75–40.25) for children in the “chronic pain” group.  Figure 2 shows the individual-level changes in total COPI scores over time.

#### 3.2.2. Item-Level COPI Scores

**Figure 3:**
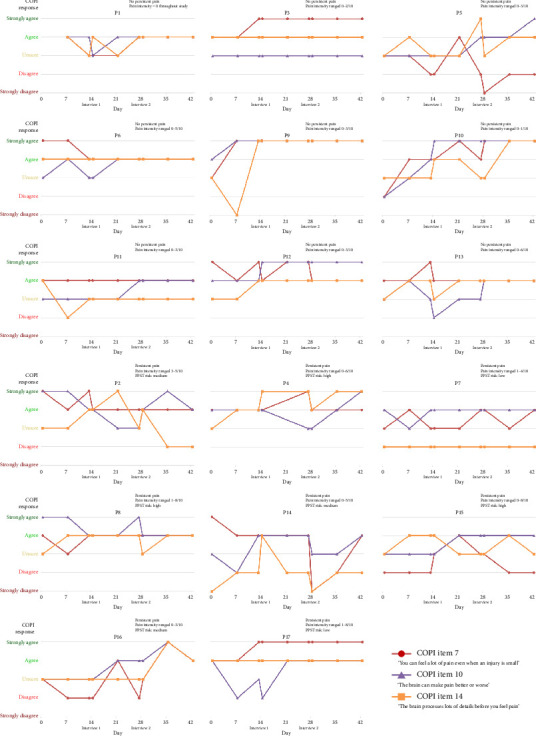
Participant-level changes in responses to COPI Items 7, 10, and 14 where change was expected only after the real PSE book in Week 4. Children with no chronic pain were presented in the top half of the figure.

Figure 3 shows item-level changes in COPI responses to Items 7, 10, and 14 for each individual participant over time. This figure includes information about the participant's chronic pain status (bottom half of figure), their PPST risk, and the range of each participant's pain intensity scores throughout the study.

For COPI Item 7, agreement ratings fluctuated by 0–3 points over time for children in the “no pain” group, with a total of 2 children “unsure” at baseline and 0 unsure at 6 weeks. Agreement ratings fluctuated by 1–4 points over time for children in the “chronic pain” group, with a total of 2 children “unsure” at baseline and 0 unsure at 6 weeks.

For COPI Item 10, agreement ratings fluctuated by 0–3 points over time for children in the “no pain” group, with a total of 5 children “unsure” at baseline and 1 unsure at 6 weeks. Agreement ratings fluctuated by 1-2 points over time for children in the “chronic pain” group, with a total of 3 children “unsure” at baseline and 0 unsure at 6 weeks.

For COPI Item 14, agreement ratings fluctuated by 0–4 points over time for children in the “no pain” group, with a total of 5 children “unsure” at baseline and 1 unsure at 6 weeks. Agreement ratings fluctuated by 0–3 points over time for children in the “chronic pain” group, with a total of 5 children “unsure” at baseline and 1 unsure at 6 weeks.

#### 3.2.3. Comparing Reports of Conceptual Change in Understanding of Pain

Perceived conceptual changes from the perspectives of an interviewer, the child participant's interview responses, and their survey responses, are presented in Table 2. The direction of the Item 7 change score matched with the interviewer's perception of conceptual change about the relationship between pain and damage 12/17 times (71%), and the change score matched with the child's perception in an interview 10/17 times (59%). The direction of the Items 10 and 14 change scores matched with the interviewer's perception of conceptual change about the influence of the brain on pain 8/17 times (47%), and these change scores matched with the child's perception in an interview 8/17 times (47%).

#### 3.2.4. Anchoring Question About Overall Conceptual Change

In the final survey, 14/17 children (*n* = 7 in each group) agreed or strongly agreed that conceptual change in understanding of pain had occurred compared to the beginning of the study and 3 children disagreed (Table 2). A slightly different 14 children improved in their total COPI scores. The Pearson correlation between the anchoring question score in Week 6 and the overall change in total COPI score was moderate (*r* = 0.48).

### 3.3. Acceptability and Usability

#### 3.3.1. Frequency of Reading

All children read the books on their own, 13 also read them with a parent and 5 with a sibling. All children read both books at least once. As of the final survey in Week 6, the frequency of self-reported reading of the two books was similar in both groups. Children with no chronic pain read “the 1^st^ book” (sham) a mean of 3.6 times and “the 2^nd^ book” a mean of 3.0 times. Children with chronic pain read these books a mean of 3.1 and 2.8 times, respectively. Eleven of the children also watched the video readings of the books that were emailed at the same time as the hardcopy books were mailed out. No children reported watching the video readings more than once.

#### 3.3.2. Qualitative Responses

Most children (80%) preferred the second book (real PSE) and thought that the books were either too short or of an appropriate length. Nearly all children (*n* = 16) liked the pictures and stated they helped with understanding. Children liked that the books were interesting and easy to understand:

*Interviewer: What did you like about the books?*

*9 yo girl (no chronic pain): That they were easy to understand and were like good for children.*

*11 yo boy (no chronic pain): I did like how it was a story … Normally when I read a lot of books about hard subjects, basically like pain, there are lots of hard words and this was easier to read.*

*9 yo girl (no chronic pain): That's pretty fun to read because it rhymes. I learned.*

Three children could not think how they might use the books or the knowledge gained in the future. Some children in both groups described using the books in the future when they were injured or felt pain, with some discussing they would use the books when checking the source of their pain:

*10 yo boy (chronic pain): Yep. Like for example … If I'm chopping something up when I'm older, like apple or something, and I'm wearing gloves or something and it goes through my glove. I would take my glove off as much as possible and see if there was actually any injury.*

*9 yo girl (no chronic pain): If I ever think there's a nail in my foot, I'm always going to check if it's not actually.*

*9 yo girl (chronic pain): … Check first before screaming.*

Some children discussed using the books in the future if they are confused about pain. Two children discussed future use of the knowledge from the sham book about rating pain. Some children in both groups said they would need to read the books again in the future after having read them once. Most felt they would be able to describe the stories to their friends and to their parents.

## 4. Discussion

Perceived conceptual change varied from the perspectives of a trained interviewer, a child's verbal responses, and their survey responses. This was found regarding both the relationship between pain and damage and about the influence of the brain on pain, over a 6-week period with a total of nine assessment time points. The two groups (with and without chronic pain) demonstrated comparable fluctuations in responses across the baseline period, after the sham book and after the real PSE book, with an overall trend of improvement suggesting the most change was attributed to the real PSE book. Children more frequently read a physical copy of the books than a video reading of the books, and qualitative responses broadly supported the acceptability and usability of these resources. All children read both books at least once, reported understanding them, and found the pictures engaging. Most children preferred the real PSE book over the sham book. Children reported liking the books because they were stories, because they rhymed, and because they could return to them multiple times.

Previous PSE literature has focused on immediate knowledge change using quizzes with correct or incorrect answers [7, 9, 10, 27, 28] rather than levels of conceptual agreement and uncertainty as per our study. A quiz approach may in fact be assessing another construct, like memory, rather than beliefs and knowledge as per the theory of conceptual change [11]. Combined survey and interview approaches have not been used in current pediatric PSE intervention research. Our findings align with other research showing that brief educational resources can likely cause lasting conceptual change [29], although larger and longer term samples are needed to confirm this finding.

Changes over time in survey responses and interview responses indicate that conceptual change is occurring. In this sample, the process of conceptual change regarding two learning outcomes about pain appeared to be very dynamic and individualized over a 6-week period. Changes occurred between all time points, suggesting that all of the following may have an influence on conceptual change: (1) PSE educational resources, (2) sham resources mentioning keywords such as “brain,” (3) completing the COPI, and (4) qualitative interviews. Some children in each group were relatively “stable” in how they responded to survey questions (Figure 3), whereas others fluctuated across the response spectrum almost every time they filled out the COPI. For example, participant 3 (P3) had conceptual change reported from the perspective of the interviewer and child in the interview and in their total COPI score (Table 2), but their responses to the most relevant items (Figure 3) appear unchanged. This highlights the challenge of trying to identify a single method to assess conceptual change in children as the most valid one. The wide variability observed in the perceptions of conceptual change from the perspectives of the interviewer, the child, and the COPI and anchoring survey responses (Table 2) is important to explore. Considering each of these trends together, using multiple approaches to assess a child's concept of pain could be useful and perhaps necessary. Given that there is no reference standard for this assessment, discrepancies between interviews and survey responses may be a useful conversation starter with children in a clinical setting to then target PSE.

Another key implication is to consider the possible role of self-reflection in causing conceptual change. Interviewers reported to the team of authors that children appeared to learn during the interviews *as they reflected* on (1) their own drawings they did during the interviews, (2) the present conversation, and (3) their independent reading of the books prior to the interviews. In addition, COPI scores changed prior to any resource being provided, so it is plausible that completing a questionnaire and participating in an interview may mediate some of the conceptual changes that could be attributed to PSE. Further research should investigate this process, and for now, clinicians should be cautious in their current interpretations of this preliminary data.

In addition, PSE rarely only includes one or two key learning outcomes as in this study; it is more common and potentially important if learnings interact with one another, that a range of learning outcomes are addressed [12, 13]. Therefore, when conceptual change education covers more than only those two ideas (namely, “pain and damage” and “the influence of the brain on pain”), it may be even more important to cross-check survey responses of conceptual change with qualitative interviewee and interviewer reports.

A key strength of this study is the SCED design with a constant sequence and blinding allowed for analysis at an individual level with a direct comparison made over time. Further to this, most studies in this field have used 1 or 2 time points to assess outcomes, whereas by having 9 time points, we demonstrated that more time points may provide valuable information about changes over time. For example, the pain intensity ratings in both groups fluctuated by up to 6–8 points out of 10 over time. Most relevant to the key findings, the inclusion of qualitative interviews allowed for children to clarify their survey responses. Limitations of the study include this being the first longitudinal item-level analysis using the COPI; any change in scores could either be a meaningful change, a nonmeaningful change, or a change due to measurement error. A longer and more stable baseline period for this SCED study is also desirable so that measured changes truly reflect real-world changes [30]. The moderate correlation (*r* = 0.48) between the anchoring question and change in COPI total scores likely reflects the challenges of assessing conceptual change with a single method. This modest association may stem from construct differences and could also partly reflect measurement error, assuming that the retrospective anchoring question is valid. This finding supports triangulation across assessment approaches, rather than favoring a single index of change. Tool validation is an ongoing process and so psychometric properties for the COPI, such as responsiveness, warrant investigation [31]. Another study limitation is that it is challenging to generalize findings of a SCED study to a broader population, and the children were recruited from a nonspecific population within one geographical region. Recruitment rates and race/ethnicity data were not collected, further limiting generalizability. Another important limitation is that the blinding of participants and assessors was imperfect and may have biased the results. The results should be interpreted with caution until well-powered studies with larger samples are conducted.

We have identified several important future research directions based on this paper. The effectiveness of PSE in children could likely be feasibly investigated in a randomized controlled trial (RCT) compared to a sham, which has not been conducted to date in either children who have persistent pain or not. A large-scale RCT powered to assess between-group differences would provide high-level evidence on the effectiveness of PSE to improve long-term behavioral participatory outcomes such as school absenteeism and presenteeism. Beyond this direction, our use of an anchoring question has raised a testable question: “do children (and possibly adults too) need to be *aware* of conceptual change?”. The final version of Zoe and Zak's Pain Hacks Book Series 1–5 has been published since this study was conducted, and various other resources for PSE have begun being developed for children [27, 32, 33], and therefore replication of the present study with newly available resources is recommended. Given that many children read the books with a parent, and this was not systematically measured or controlled for, future research could explore the specific role of parent–child co-reading. Our finding is that children returned to books more often than videos suggest that a reading resource may have increased learning utility and impact, potentially via learning repetition, and this could also be studied further.

## 5. Conclusions

Understanding about the relationship between pain and damage, and about the influence of the brain on pain, generally improved throughout the study. Perceived conceptual change is different for different observers and methods of reporting. Concept of pain is a dynamic construct and specific ideas may be amenable to conceptual change via the provision of targeted educational resources. All children read both books at least once. Most children preferred the real PSE book over the sham book.

## Figures and Tables

**Table 1:** Demographics of child participants.

Characteristics	No chronic pain group *n* = 9, *n* (%)	Chronic pain group *n* = 8, *n* (%)
Gender (female)	4 (44)	4 (50)
Age (years)		
8	0 (0)	0 (0)
9	5 (56)	3 (38)
10	3 (33)	2 (25)
11	1 (11)	3 (38)
12	0 (0)	0 (0)
Index of relative socioeconomic disadvantage, SEIFA^∗^ decile/10		
1	0 (0)	0 (0)
2	0 (0)	0 (0)
3	1 (11)	0 (0)
4	0 (0)	0 (0)
5	0 (0)	0 (0)
6	0 (0)	1 (13)
7	0 (0)	2 (25)
8	1 (11)	1 (13)
9	4 (44)	2 (25)
10	3 (33)	2 (25)
Chronic pain		
Yes	0 (0)	8 (100)
(i) Duration (open-ended question)		
0-1 year		1 (13)
1-2 years		2 (25)
3–5 years		4 (50)
6–10 years		1 (13)
(ii) Location (tick all that apply)		
Head		1 (13)
Face or jaw		2 (25)
Shoulder or neck		2 (25)
Chest		1 (13)
Arm or hand		3 (38)
Abdomen or stomach		1 (13)
Back		2 (25)
Leg or foot		7 (88)
Other location(s)		4 (50)
(iii) PPST risk		
Low		1 (11)
Medium		3 (33)
High		4 (44)
In the past 3 months, have you had any of the following? (tick all that apply)		
Pain	4 (44)	8 (100)
Injuries	1 (11)	3 (38)
Medicine for pain	3 (33)	7 (88)
Have visited a health professional about pain	3 (33)	6 (75)
Surgery	0 (0)	0 (0)
No, none of these	5 (55)	0 (0)

*Note:* For pediatric pain screening tool (PPST) [22], total scores of 0–2 are considered “low risk” and psychosocial subscale scores of 3 or more are considered “high risk.”

^∗^SEIFA = socioeconomic indexes for areas, Australia, 2016.

**Table 2:** Perceived conceptual changes from the perspectives of the interviewer, the participants' verbal responses, and their survey responses.

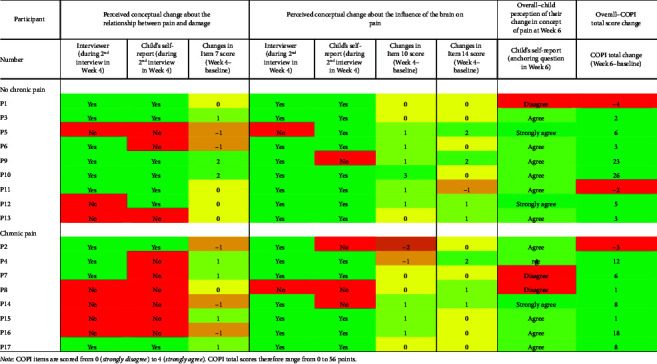

## Data Availability

Data underlying this study are available from the corresponding author upon reasonable request.

## Supporting Information

Supporting information 1 contains the qualitative interview methods, codebook, and topic summaries relating to the purpose and definition of pain.
