# Supplementary material for: Targeting Pain Science Education With a Children's Book: A Single Case Experimental Design (SCED) Study With a Sham Comparison
Source: Pain Res Manag. 2025 Nov 5;2025:7548771. doi: 10.1155/prm/7548771 (PMC12611469; doi:10.1155/prm/7548771)
Supplement: Supporting Information — Additional supporting information can be found online in the Supporting Information section. [file 7548771.f1.docx]

**Supplementary material 1:** Qualitative interview methods codebook and topic summaries relating to the definition of pain, the purpose of pain, and the mechanisms underpinning pain.

**Methods**

Children were invited to draw what they think of when they hear the word ‘pain’, and interviewers referred to these drawings to prompt explanations. Coders used the child’s verbal explanations when applying the codebook.

To minimize potential bias because JWP is the author of the intervention book (and sham book), he was not involved in the interviews or thematic analysis. Audio recordings were transcribed by JN and EM, research assistants working as pediatric physiotherapists, verbatim. JN and EM independently checked transcripts for accuracy. Realist evaluation guided the qualitative design, data collection and analysis [34]. Realist evaluation sits between the philosophical underpinnings of positivism and constructivism. Codebook thematic analysis was used to align with such underpinnings as well as the applied nature of the research[35, 36]. A predetermined coding frame was developed based on empirical and theoretical work in conceptual change theory and the biopsychosocial model of pain[37]. Pilot data (8 baseline and 8 follow-up pilot interviews) informed refinement of the coding frame. Each interview was independently coded by two of three researchers (ET, JN and EM) who considered each line of text or participant response to the interview questions. The researchers met regularly to discuss coding and ensure consistent application of the codebook. NVivo software version 12.0 facilitated analysis. Resulting codes were developed into categories as they related to the coding frame, allowing flexibility for the coding frame to iteratively develop based on the data. Similar categories were then grouped to form initial topic summaries which was scrutinized by the team of authors which includes pediatric clinical psychologists, physiotherapists, academics and methodological experts, in two meetings during the analytical process and one final discussion to confirm agreement.

**Codebook**

| Initial topic summaries | Initial categories |
| --- | --- |
| Definition of pain: ‘what’ | Pain from an injury/disease |
|  | Biological contributors |
|  | Psychological contributors |
|  | Social contributors |
| *Second interview only* | Change in definition |
| Purpose of pain: ‘why’ | Alerts that something is wrong |
|  | Avoid further damage |
| *Second interview only* | Change in understanding the purpose of pain |
| Mechanisms underpinning pain: ‘how’ | Pain is about protection, not damage |
|  | Nerve pathways/messages eg. Eyes make ‘phone call’ to brain detecting danger |
|  | Many variables influence the brain |
|  | Influence of the brain on pain eg. brain makes pain worse or better |
| *Second interview only* | Change in understanding mechanisms |
| Acceptability and usability | What was good about the books |
|  | What they didn’t like about the books |
|  | Length of books |
|  | Pictures |
|  | Suggestions to improve the books |
|  | Future use of the books |
|  | Book preference (1 or 2) |

**Topic Summary 1: Definition of pain**

Children found it difficult to define pain, particularly those without chronic pain, who reflected on memories to describe pain. Children associated pain with an injury, while a few nominated other causes such as a disease or illness:

*Researcher: So you’ve drawn a scratch on the leg. Why have you drawn that today?*

*9yo boy (no chronic pain): Because it’s just like pain. You feel pain when you get scratched.*

Few children described social contributions to pain. Of those that did, responses included reference to emergency services: *“ambulance or fire truck”*, and a cross or danger symbol. Beyond the brain (see below for further details), the only additional reference to biological contributions to pain was blood.

Children described pain as feeling achy, burning, sore, stinging/tingling or stabbing. There were a variety of reactions to pain discussed, most of which were negative emotions such as sad, angry, and confused.

**Topic Summary 2: Purpose of pain**

Only one child identified that pain indicates that something is wrong:

*9yo boy (no chronic pain): “So it’s telling you that your body needs to be repaired and you need to stop doing what you’re doing, cause it’s making it hurt.”*

Some discussed that pain helps to avoid further damage and without pain, more damage could result:

*Researcher: What do you think would happen if we couldn’t ever feel pain?*

*10yo girl (chronic pain): (8 sec pause) You would hurt yourself a lot more often cause ummm... You wouldn’t be able to feel pain and you wouldn’t notice it.*

When first prompted, most children stated that if a person could not feel pain, that would be positive as people would not feel sad from pain, and there would be less need for medical professionals and hospitals.

*Researcher: What do you think would happen if we couldn’t ever feel pain?*

*10yo girl (no chronic pain): Well, people would feel happier, and it would be like nice to not have pain.*

A few children were not sure what would happen if pain did not exist, initially suggesting they were unsure of the purpose of pain.

Most children thought it is possible to have pain without damage, though a couple were unsure. Of those that answered it is possible to have pain without damage, all described the pain as still being real. Children were unable to elaborate on this answer to provide an explanation about how it is possible to have pain without damage.

Children were mixed in their responses about whether it is possible to have damage without pain. Some were able to identify this when first asked:

*Researcher: Do you think you can have damage in your body, like an injury, and not feel any pain?*

*9yo girl (chronic pain): Well. Yes and no. Because apparently once I broke my toe but I didn’t know.”*

While others did not think it was possible:

*10yo girl (no chronic pain): (6 sec pause) I don’t think so, because usually the injury is what describes pain.”*

When further prompted about the possibility of being distracted when in pain (see below for more information), all children were able to identify that it could be possible to have damage but not experience pain, or to reduce the pain.

**Topic Summary 3: Mechanisms underpinning pain**

While most children were unsure whether it is possible to have damage without pain (see above) when prompted, all were able to respond that it is possible to be distracted from pain, which could mean the damage is still present, but the pain lessened:

*9yo girl (chronic pain): Well like. [3 sec pause] If I’m like enjoying something, it feels like it doesn’t like.. The pain doesn’t completely go away but it like has less, is like less sore and it distracts me from the pain...*

As such, most children thought that the brain can help to reduce pain. Children’s responses were mixed as to whether the brain can make pain worse.

*9yo girl (chronic pain): It can make you feel better because it can distract you, and it can make it feel worse cause like…. (10 sec pause) I don’t know.*

*10yo boy (chronic pain): It can make it worse because like if your brain makes you distracted you can take your mind off the pain but if your brain can’t get distracted and it’s really tense and worried and stressed it would probably make it worse.*

Children were able to identify and to some degree discuss the influence of the brain on pain. While few children offered the brain’s role in pain as a response to the question “what does the brain do?”, with further prompting, all were able to describe a link between the brain and pain:

*10yo girl (chronic pain): Well, it… The pain... You would feel it and then you would think it and then you would feel the pain because you brain is controlling it and because it hurt your brain would tell you to say “OW”.*

Some children were not able to articulate their understanding of the nerves, with some stating that pain travels through the veins. When prompted to think about messages sent to the brain, most were able to discuss the link between pain, nerves and the brain:

*9yo girl (chronic pain): [21 sec pause – using picture to draw] Can you see the blue? Like the hand and the mouth? Like up there, and their mouth gets messages from the brain that it’s told by the nerves, that it got like hurt, I guess?*

*9yo girl (no chronic pain): So, if the stove is there, the pain goes up to the hand and that processes up to the brain and then the brain sends out messages to the other parts and tells them to worry and it makes pain worse or better. And then all that pain processes up to the head, and then processes it to the mouth and it can share it.*

*Researcher: And how do those messages travel? Do they travel through anywhere in particular?*

*9yo girl (no chronic pain): Ummm through nerves and stuff.*
